# Comorbidities in a Cohort of 66 Patients With Psoriatic Arthritis Mutilans—Results From the Nordic PAM Study

**DOI:** 10.3389/fmed.2021.629741

**Published:** 2021-02-04

**Authors:** Josephine Mistegård, Bjorn Gudbjornsson, Ulla Lindqvist, Leena Laasonen, Leif Ejstrup, Mona Ståhle, Lars Iversen

**Affiliations:** ^1^Department of Dermatology, Aarhus University Hospital, Aarhus, Denmark; ^2^Faculty of Medicine, Centre for Rheumatology Research, Landspitali University Hospital, University of Iceland, Reykjavik, Iceland; ^3^Department of Medical Sciences, Rheumatology, Uppsala University, Uppsala, Sweden; ^4^Helsinki Medical Imaging Center, Helsinki University Central Hospital, Helsinki, Finland; ^5^Department of Rheumatology, Odense University Hospital, Odense, Denmark; ^6^Dermatology Division, Department of Medicine, Karolinska Institutet, Stockholm, Sweden

**Keywords:** psoriasis, arthritis, mutilans, comorbidity, multinational study

## Abstract

**Objective:** Psoriatic arthritis mutilans (PAM) is the most severe phenotype of psoriatic arthritis due to excessive bone erosion causing joint destruction and decreased functional capacity. The aim of this study was to investigate the prevalence of comorbidities among patients with PAM and the association between comorbidities and joint involvement.

**Methods:** A total of 66 patients aged ≥18 years from the Nordic countries with past or present psoriasis along with at least one mutilated joint were included in the present study.

**Results:** The median number of comorbid conditions per patient was 1 [interquartile range (IQR) 0–2] and 16.7% reported three or more comorbidities. The most frequent comorbidity was hypertension (36.4%). The median number of mutilated joints per patient was 3 (IQR 1–8.3; range 1–38).

**Conclusion:** Two thirds of the patients with PAM reported comorbid conditions and the most frequent was hypertension which affected more than a third of the patients. However, this study was unable to detect any association between comorbidities and the severity of PAM.

## Introduction

Psoriatic arthritis (PsA) is an inflammatory musculoskeletal disease affecting 20–30% of patients with psoriasis (1–3). In the majority of patients with PsA, skin affection precedes arthritis by several years (4). PsA is characterized by synovial and entheseal inflammation along with extraarticular manifestations, such as nail lesions and uveitis (4, 5). Variable clinical presentations have given rise to five PsA subtypes: arthritis in the distal interphalangeal joints, asymmetric oligoarthritis, symmetric polyarthritis, axial spondyloarthritis, and psoriatic arthritis mutilans (PAM) (6). Any of these subtypes may be present in different combinations and the disease pattern may change over time (4, 7).

PAM is the most severe clinical subtype of PsA and is characterized by digital telescoping termed “opera-glass finger,” resulting in loss of function of the affected joint (6). The study of PAM has been impeded by the lack of internationally accepted classification criteria. However, attempts have been made in order to define PAM (8). Due to inconsistent classification criteria, reports on the prevalence of PAM vary widely with estimates ranging from 0.6 to 21% among patients with PsA (4, 6, 9–13). Meanwhile, the Nordic PAM Study reported a prevalence of nearly four cases per million inhabitants in the Nordic population (14). However, arthritis mutilans, may also occur along with rheumatoid arthritis, chronic reactive arthritis, and juvenile arthritis (15). Most previous reports of PAM are serial case reports and only few reports have included more than ten patients (3, 14, 16–19).

The pathogenesis of the different PsA phenotypes is still not fully understood. Genetic factors associated with psoriatic disease have become more clearly defined and may contribute to our understanding of the different clinical presentations of PsA (5, 20). Moreover, psoriatic disease is associated with a number of comorbidities. For example, metabolic syndrome is more prominent in patients with PsA than in the general population (21, 22). Also, the risk of cardiovascular disease (CVD) is increased among patients with PsA (22, 23). Shared inflammatory pathways, proinflammatory cytokine release and adhesion molecule expression may contribute to this association. Tumor necrosis factor (TNF)-α has been associated with endothelial dysfunction, and interleukin (IL)-17A has been associated with increased expression of adhesion and pro-inflammatory molecules associated with CVD (24, 25). Furthermore, it is well-established that the risk of mental comorbidities, such as depression and anxiety is higher among patients with psoriasis than in the general population (26–28). This may, in part, be caused by the psychosocial difficulties associated with having psoriasis but may also be related to overlapping mechanisms. The level of the proinflammatory cytokines IL-1 and IL-6 are increased in patients with both PsA and depression, indicating involvement of the same inflammatory process in both diseases (26). In addition, other inflammatory disorders, such as chronic obstructive pulmonary disease (COPD) and inflammatory bowel disease (IBD) are more prevalent among patients with PsA than among the general population (29–32).

Given its severe rheumatic manifestation, PAM may be associated with a higher inflammatory burden than other PsA phenotypes. The aim of this study was to investigate the prevalence of comorbidities among patients with PAM in the Nordic PAM Study and to explore the association between these comorbidities and joint involvement.

## Methods

**Figure 1:**
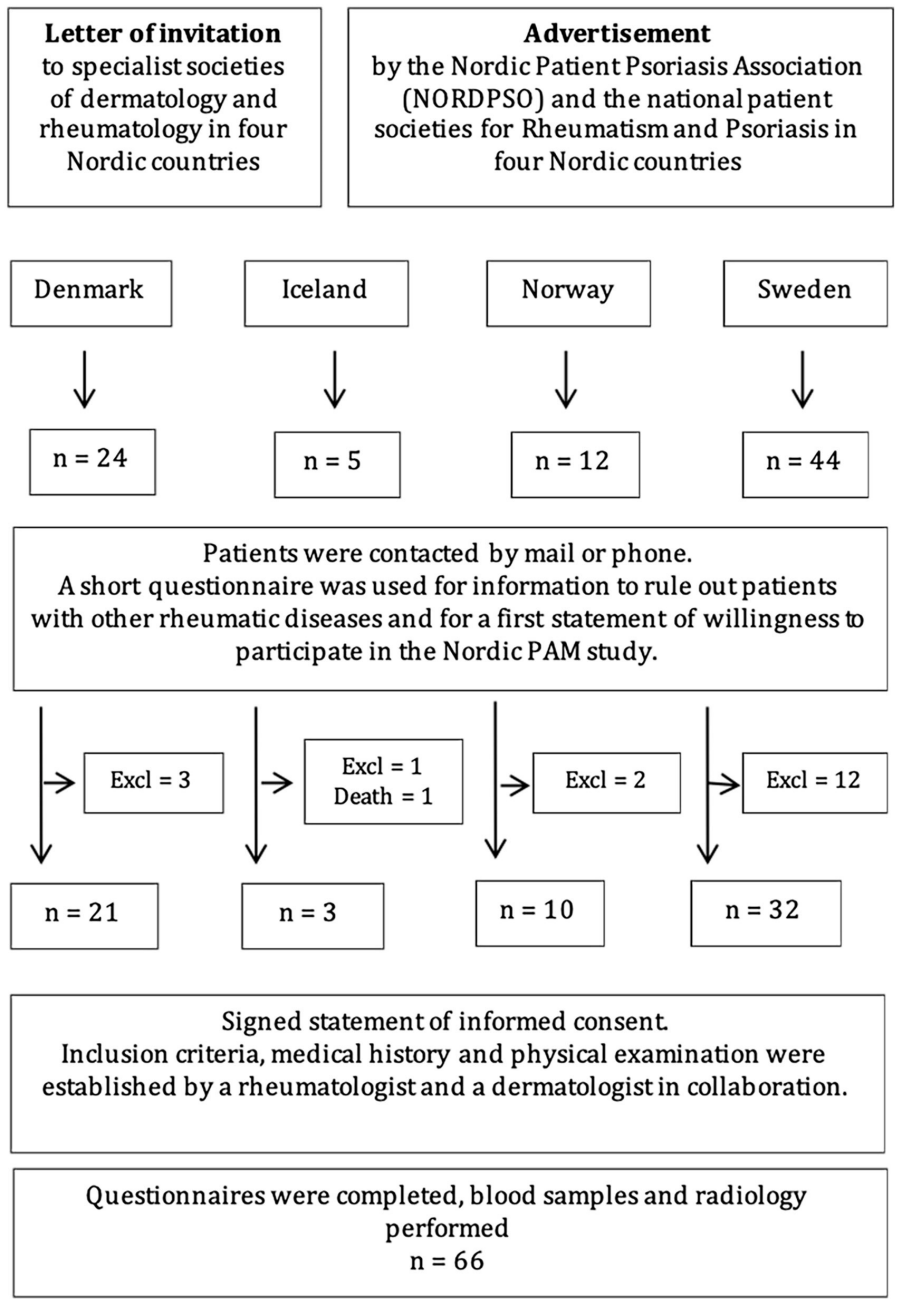
STROBE flowchart of the recruitment and inclusion process.

Patients were identified as part of The Nordic PAM Study initiated in 2009 to investigate PAM in the Nordic countries. Data collection has previously been described in detail (14, 17, 33–35). The study included patients from Denmark, Iceland, Norway and Sweden. Patients were identified in collaboration with specialist societies of dermatology and rheumatology and patient associations in each country. Included patients met the following inclusion criteria: (I) able and willing to give written informed consent; (II) age ≥18 years; (III) past or present psoriasis (skin or nails) diagnosed by a dermatologist; (IV) presence of clinical arthritis of the PAM type verified by a rheumatologist; and (V) radiological findings of severe erosive arthritis, e.g., pencil-in-cup or gross osteolysis of the bones. Patients meeting any classification criteria for any other rheumatic joint disease were excluded from the study. At present 70 patients have been included in the study. However, four patients were excluded from the present analysis due to missing information on joint involvement, leaving a cohort of 66 patients with PAM. The current inclusion is depicted in Figure 1 (14). The latest patient was included in April 2014. Cohort characteristics have been described previously and patient demographics of the present cohort are presented in Table 1 (14, 33, 35).

**Table 1:** Demographics of patients included in the Nordic PAM Study.

**Country**	**Number of patients**	**Age (years) Mean ± SD (range)**	**Gender, male *n* (%)**	**Gender, female *n* (%)**
Denmark	21	55.14 ± 10.75 (41–75)	10 (48)	11 (52)
Iceland	3	70.67 ± 10.07 (63–83)	3 (100)	0 (0)
Norway	10	52.90 ± 9.96 (44–79)	6 (60)	4 (40)
Sweden	32	60.09 ± 11.18 (42–93)	13 (41)	19 (59)
Total	66	57.91 ± 11.32 (41–93)	32 (48)	34 (52)

### Clinical Examination and Data Collection

All patients who met the inclusion criteria underwent clinical evaluation by board-certified specialists in rheumatology and dermatology. The extent of joint involvement was assessed according to the 66/68 American College of Rheumatology (ACR) joint count for swelling and for tenderness. This joint count includes both the proximal interphalangeal (PIP) and the distal interphalangeal (DIP) joints of hands and feet. The presence of joint deformities, joint mutilation, dactylitis, and enthesitis was registered. A joint was considered mutilated in the presence of both clinical manifestation of PAM and joint destruction characterized by osteolysis on X-ray evaluation. Joints were radiographed at inclusion if the most recent previous radiographs had been made more than 3 months prior to inclusion. X-rays were evaluated by a radiologist and the findings have been reported elsewhere (34, 35). Information on current and past history of comorbid medical illness was obtained by the investigators as part of a semi-structured interview. Listed comorbidities included cancer, diabetes mellitus, COPD, IBD, cerebral insult, hypertension, acute myocardial infarct, heart failure, other autoimmune disease, infectious disease requiring hospitalization, and others. For each patient, the type, number, onset date, and potential current symptoms and required treatment were registered for the reported comorbidities. The investigators reviewed the patients' medication list in order to verify the reported comorbidities.

### Data Analysis

All patient data were coded at inclusion. Continuous data were expressed as mean ± standard deviation (SD) for normally distributed data, and non-normally distributed data were expressed as median with interquartile range (IQR). Normality was assessed by plotting histograms and performing the D'Agostino-Person normality test. Categorical variables were analyzed using descriptive statistics and reported as frequency (*n*, %). The descriptive statistical analysis was done in Microsoft Excel 2019. Fischer's exact test was performed in 2 × 2 tables to investigate the association between the number of comorbid conditions and the number of affected joints. This test and the D'Agostino-Person test were carried out in GraphPad Prism 8.

### Ethical Issues

Written informed consent was obtained from all study participants. The bioethics committee and data protection authorities of all four countries approved the study protocol.

## Results

### Joint Involvement

**Figure 2:**
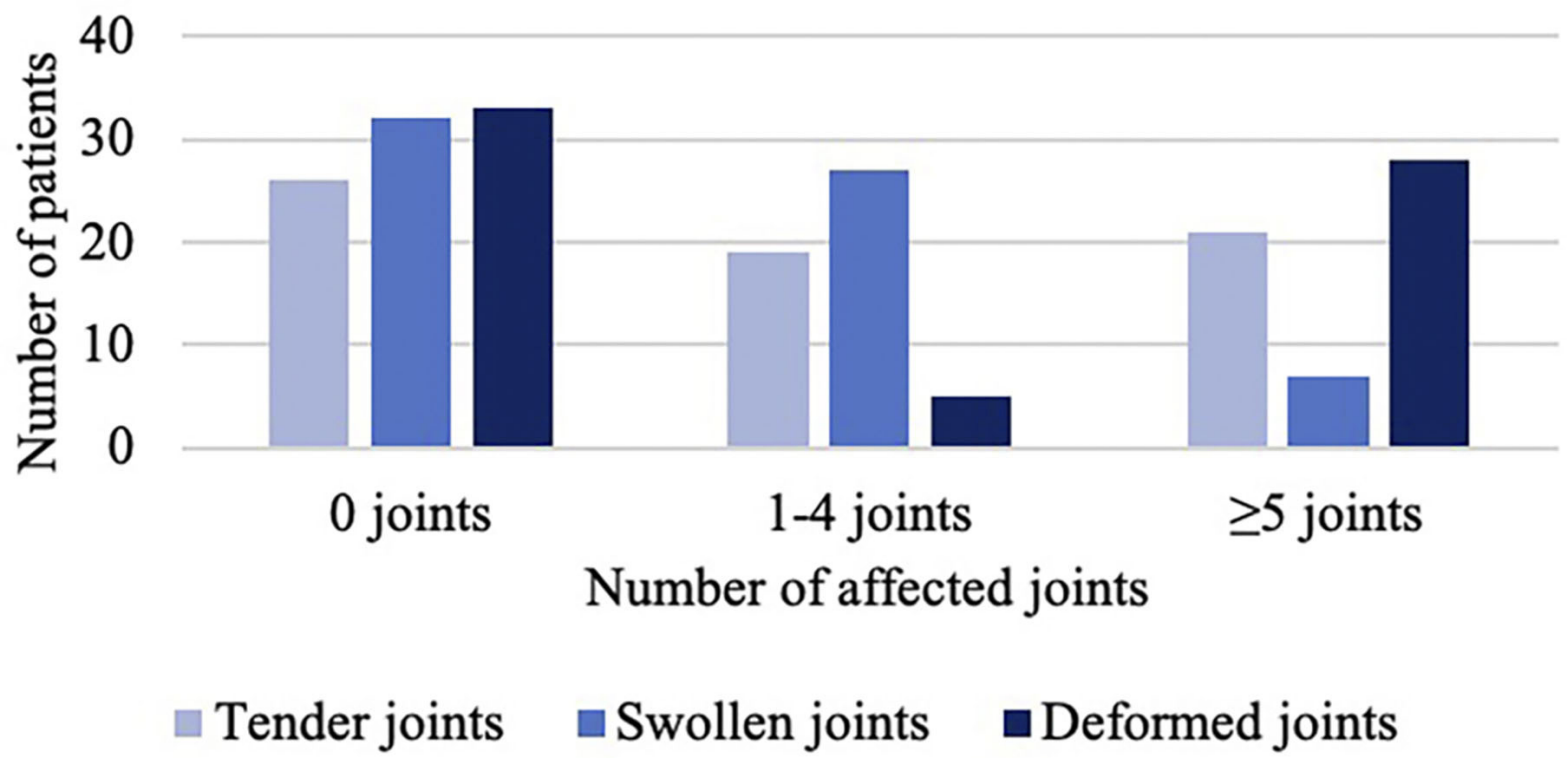
Number of tender, swollen, and deformed joints.

**Figure 3:**
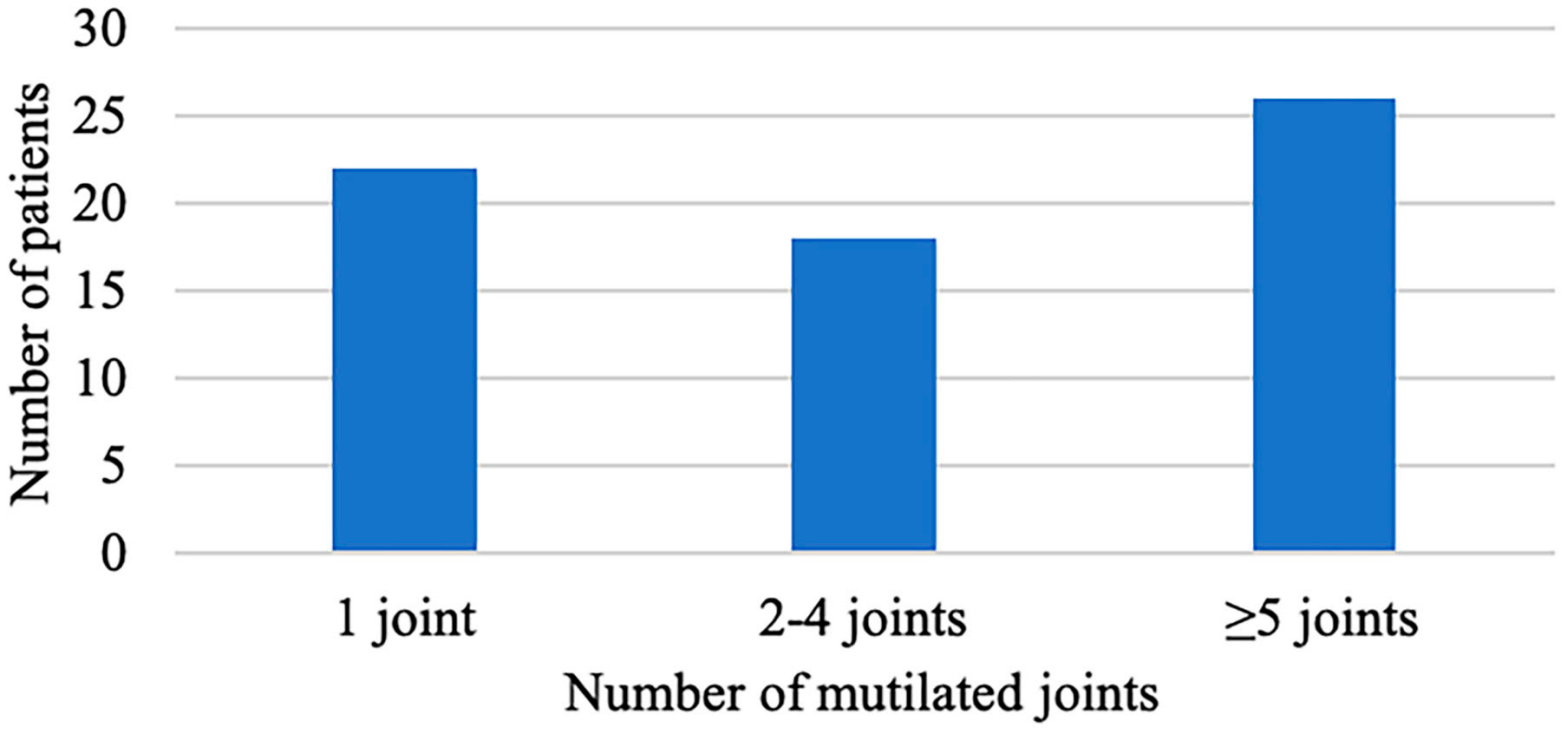
Number of mutilated joints.

The median number of tender joints per patient was 1 (IQR 0–7) ranging from 0 to 35, and the median number of swollen joints per patient was 1 (IQR 0–3) ranging from 0 to 16. The median number of deformed joints per patient was 0.5 (IQR 0–12.5) ranging from 0 to 59 (Figure 2). In accordance with the inclusion criteria, all included patients had a minimum of one mutilated joint. The median number of mutilated joints per patient was 3 (IQR 1–8.3) ranging from 1 to 38, with 39.4% of patients presenting five or more and 19.7% presenting ten or more mutilated joints (Figure 3).

### Comorbidities

**Figure 4:**
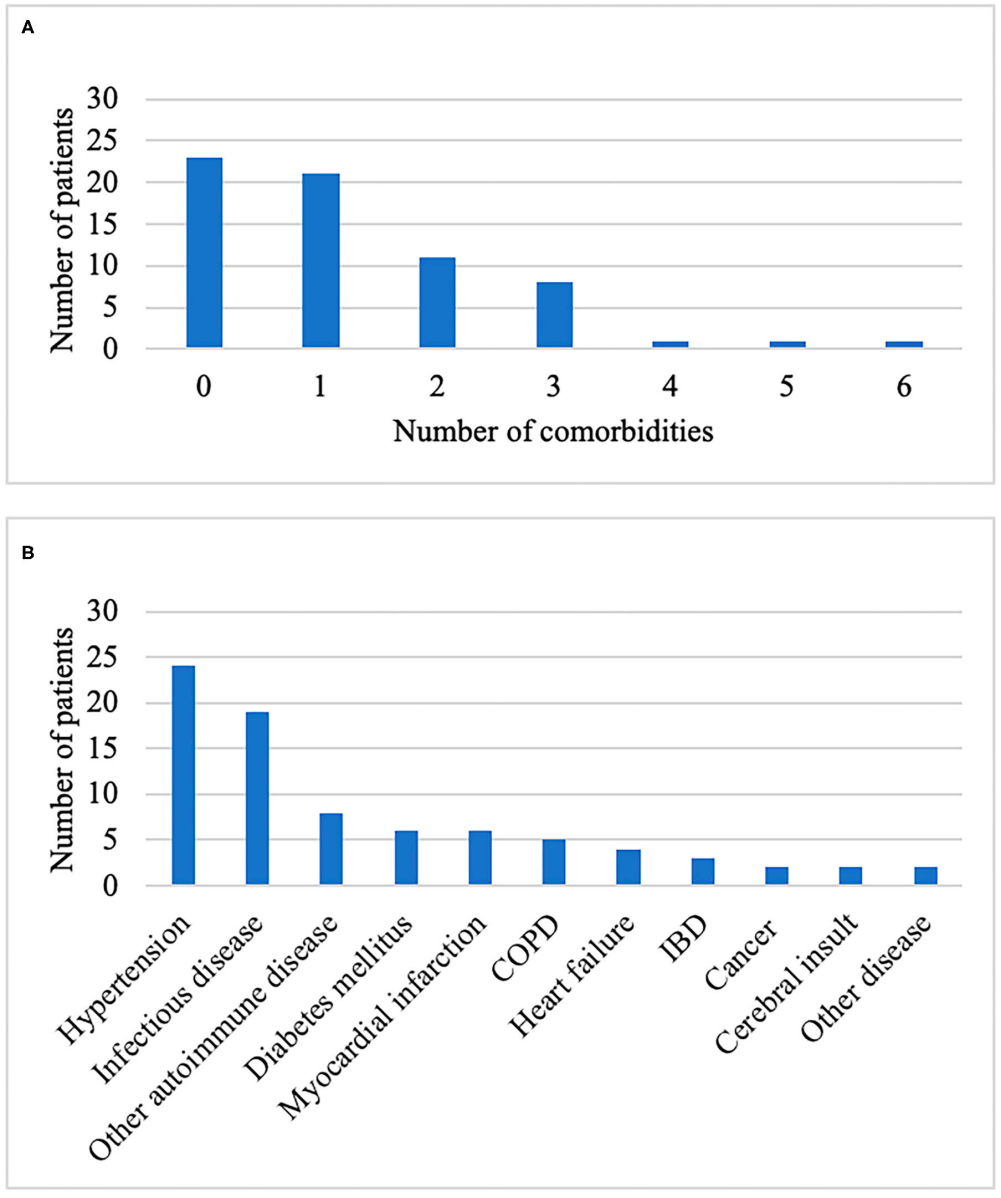
Comorbidities among participants in the Nordic PAM study (*n* = 66). **(A)** Number of comorbidities per participant. **(B)** Type of comorbidity. COPD, chronic obstructive pulmonary disease; IBD, inflammatory bowel disease.

Comorbid conditions were reported by 65.2% of the participants. The median number of self-reported comorbid conditions per patient was 1 (IQR 0–2). A current or past history of two or more comorbidities was reported by 33.3% of the patients, and 16.7% reported three or more comorbidities (Figure 4A). Having two or more comorbidities was equally frequent among men and women (11:11). The most frequent comorbidity was hypertension requiring medical treatment (36.4%), followed by infectious diseases requiring hospitalization (28.8%) and other autoimmune diseases (12.1%) (Figure 4B).

The most frequently reported infectious diseases requiring hospitalization were pneumonia (*n* = 5) and skin infection due to staphylococci (*n* = 3). Autoimmune diseases included thyroid disease (*n* = 5), uveitis (*n* = 2), and lung fibrosis (*n* = 1). The reported cases of cancer were non-melanoma skin cancers (*n* = 2).

### Correlation Between Comorbidity and the Severity of the Joint Disease

Fischer's exact test was performed to evaluate the probability of patients having one or more comorbidities in case of one or more tender joints, swollen joints, or deformed joints. Furthermore, Fisher's exact test was used to evaluate the likelihood of patients having one or more comorbidities in case of more than two mutilated joints. This test found no correlation between the number of comorbidities and the number of affected joints (Supplementary Figures 1–4).

## Discussion

The Nordic PAM Study offers a unique opportunity to further investigate this rare and disabling disease, since this is presently one of the largest cohorts of patients with PAM worldwide. To the authors' knowledge, the present study is the first study to investigate the prevalence and profile of comorbidities among patients suffering from PAM. The results are in line with previously published studies describing the comorbidity profile among patients with PsA.

Recent studies of comorbidities among patients with PsA reported a median of two comorbid conditions per patient. More than a third of these patients had three or more comorbidities and some patients reported up to 12 comorbid conditions (36, 37). In the present study of patients with PAM, the median number of self-reported comorbid conditions per patient was one comorbidity and some patients reported up to six comorbidities. One third of the patients with PAM reported a current or past history of two or more comorbidities, and one fifth reported three or more comorbidities. PsA has an impact on quality of life, which is directly related to disease activity and structural damage of the joints. However, increased comorbidity burden has also been independently associated with decreased functional capacity. This considered, detection and control of comorbid conditions among patients with PsA may serve to improve quality of life in this group of patients (38).

Hypertension is frequent among patients with PsA and similar to the frequency found among patients with PAM in the present study (36, 37). This is consistent with other studies suggesting an increased prevalence of metabolic syndrome among patients with PsA compared with the general population (21, 22, 39, 40). Frequencies of diabetes mellitus and CVD are also similar between patients with PAM and patients with PsA in general (36, 37). Yet, heterogeneity in the definition and categorization of comorbidities hampers direct comparison between studies. Since the preparation of the Nordic PAM Study protocol, it has been well-established that the risk of mental comorbidities, such as depression and anxiety is higher among patients with psoriatic disease than among the general population (26–28). These are some of the most frequent comorbidities among patients with PsA, affecting approximately one third of the patients (36, 37). Data on mental illness were not collected in the Nordic PAM Study, which may very well have contributed to the relatively low reported number of comorbidities in the Nordic PAM Study. PAM is the most severe PsA phenotype and seriously affects social functioning and quality of life. Thus, mental illness may be expected more frequently among patients with PAM than among patients suffering from other PsA subtypes (33).

As described initially, many studies have indicated that the variable clinical presentations of psoriatic disease may be related to the severity of the inflammatory burden. In this context, skin severity has been described to be correlated with joint activity; thus, patients with higher skin severity are two times more likely to have increased joint involvement (41). Moreover, comorbid conditions in the form of depression and anxiety along with hypertension and diabetes mellitus are described more frequently among patients suffering from both psoriasis in the skin and PsA than among patients with only psoriasis in the skin (27, 28). In addition, studies have found that cardiovascular risk factors are more strongly associated with severe psoriasis than with mild psoriasis (28, 42). In the present study, we analyzed the correlation between the severity of the joint affection and comorbidities by applying Fisher's exact test. This test found no association either between the number of comorbidities and chronic alterations in the form of mutilated or deformed joints or between the number of comorbidities and present inflammatory alterations at the time of inclusion in the form of tender or swollen joints. Hence, this study was unable to confirm the association that other studies have suggested.

There are still no internationally accepted diagnostic criteria for this rare variant of PsA. Therefore, an inclusion criterion in the Nordic PAM Study was the presence of both clinical manifestation of PAM and confirmation of joint destruction characterized by osteolysis on X-ray evaluation.

Information on comorbidities was collected by the investigators as part of a semi-structured interview and verified through a review of the patients' medication list. The investigators registered the type, number, onset date, and potential current symptoms and required treatment for each reported comorbidity. Access to all parts of the patients' electronic medical file was not permitted in the study protocol. The use of a semi-structured interview may in this case have caused underreporting of comorbidities and thereby underestimation of the number of comorbidities among patients with PAM.

In conclusion, comorbidities are common among patients with PAM as two thirds of the participants in the Nordic PAM Study reported comorbid conditions. The most frequent comorbidity was hypertension which affected more than one third of the participants. This knowledge is important for diagnostic and therapeutic purposes alike, as knowledge of comorbidities may serve to ensure sufficient treatment and improved prognosis for patients suffering from PAM. The present study found no association between severity of joint affection and number of comorbidities. Future studies may focus on further evaluation of the significance of the inflammatory burden in the pathogenesis of the various PsA phenotypes.

## Data Availability Statement

The data analyzed in this study is subject to the following licenses/restrictions: the datasets contain personally identifiable information and has therefore not been published in accordance with Danish legislation. Requests to access these datasets should be directed to Lars Iversen, lars.iversen@clin.au.dk.

## Ethics Statement

The study protocol was approved by the Bioethics Committee and Data Protection Authorities of Denmark, Iceland, Norway and Sweden. The study participants provided their written informed consent to participate in this study.

## Author Contributions

JM, BG, UL, LL, LE, MS, and LI contributed to the design and implementation of the research, to the analysis of the results, and to the writing of the manuscript. All authors contributed to the article and approved the submitted version.

## Conflict of Interest

The authors declare that the research was conducted in the absence of any commercial or financial relationships that could be construed as a potential conflict of interest.
