# Supplementary material for: Comorbidities in a Cohort of 66 Patients With Psoriatic Arthritis Mutilans—Results From the Nordic PAM Study
Source: Front Med (Lausanne). 2021 Feb 4;8:629741. doi: 10.3389/fmed.2021.629741 (PMC7889950; doi:10.3389/fmed.2021.629741)
Supplement: Supplementary file 1 [file Table_1.DOCX]

Supplementary Material

# Supplementary Figures and Tables

## Supplementary Figures


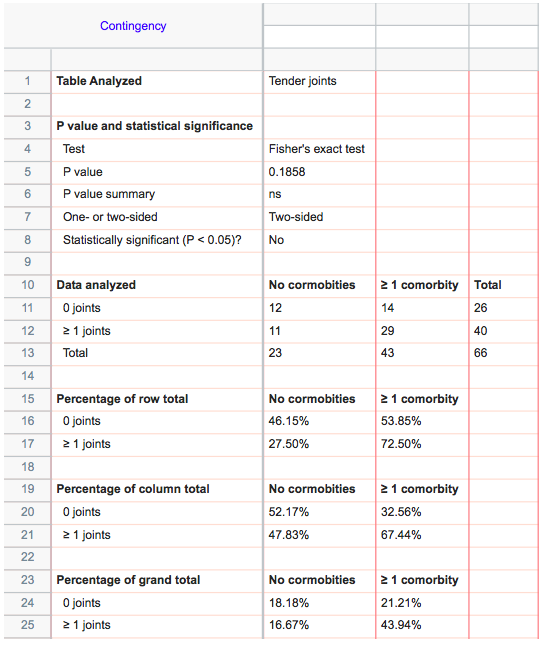


**Supplementary Figure 1.** Statistical test on association between the number of comorbidities and the number of tender joints.

**
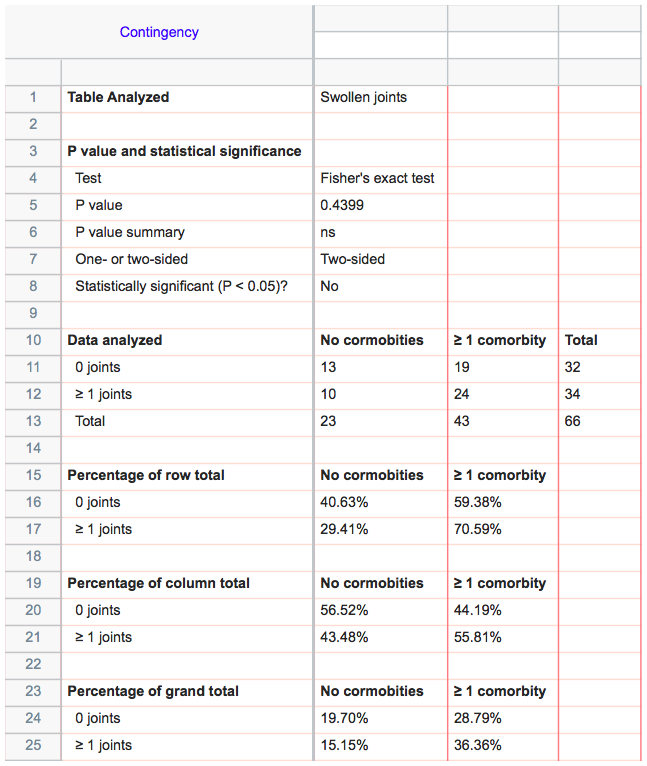
**

**Supplementary Figure 2.** Statistical test on association between the number of comorbidities and the number of swollen joints.

**
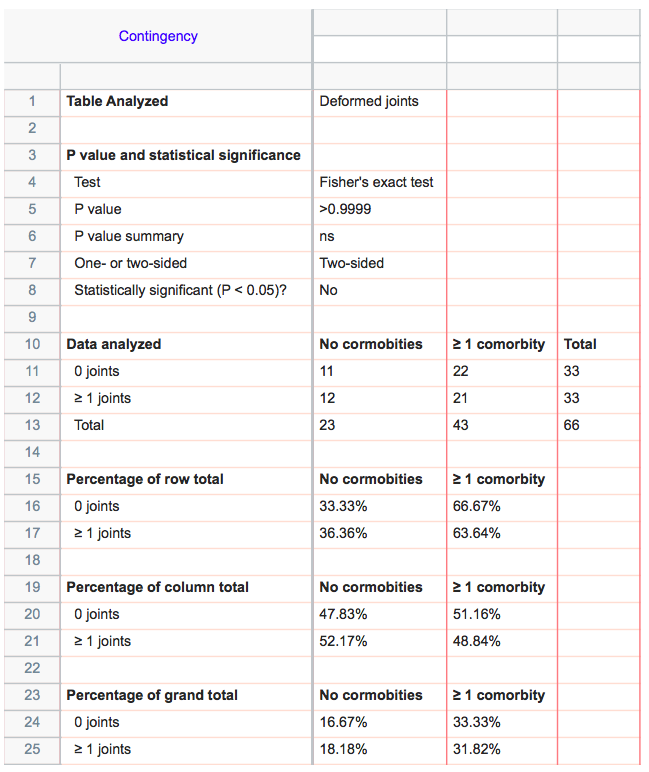
**

**Supplementary Figure 3.** Statistical test on association between the number of comorbidities and the number of deformed joints.

**
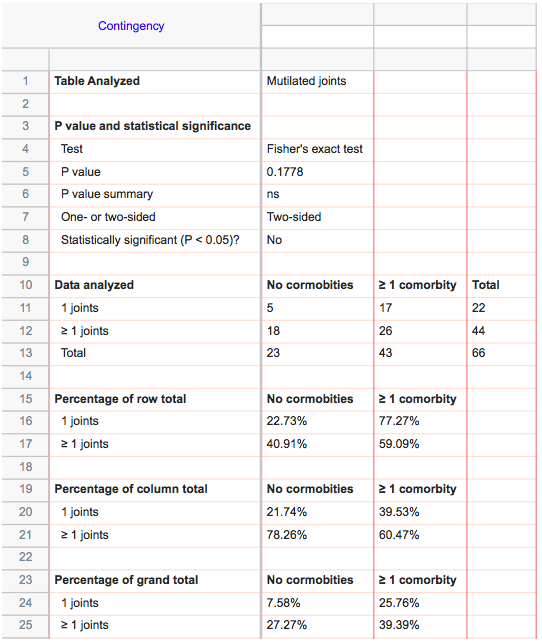
**

**Supplementary Figure 4.** Statistical test on association between the number of comorbidities and the number of mutilated joints.
